# ADHD symptoms and efficient learning strategies in college students: the parallel mediating roles of motivation and mind-wandering

**DOI:** 10.3389/fpsyg.2026.1830674

**Published:** 2026-10-07

**Authors:** Li Yang, Ximing Wang, Anni Liu, Xinni Li, Weiqi Peng, Cheer Muer

**Affiliations:** 1 School of Mental Health, North Sichuan Medical University, Nanchong, China; 2 School of Management, North Sichuan Medical University, Nanchong, China; 3 School of Clinical Medicine, North Sichuan Medical University, Nanchong, China; 4 School of Basic Medical Sciences and Forensic Medicine, North Sichuan Medical University, Nanchong, China

**Keywords:** ADHD symptoms, learning motivation, learning strategies, mind-wandering, the role of mediation

## Abstract

A questionnaire survey was conducted among college students from a university in Sichuan Province to examine the relationship between ADHD symptoms and the use of efficient learning strategies, with a focus on the parallel mediating roles of learning motivation and mind-wandering. Of the 919 questionnaires distributed, 730 valid responses (*N* = 730) were retained for analysis. Structural equation modeling with bootstrap confidence intervals indicated that learning motivation and mind-wandering acted as parallel mediators in the relationship between ADHD symptoms and strategy use, with both learning motivation and mind-wandering showing negative indirect effects (learning motivation: *β* = −0.130, 95% CI [−0.196, −0.061]; mind-wandering: *β* = −0.107, 95% CI [−0.198, −0.027]). The direct effect of ADHD symptoms on strategy use was non-significant (*β* = −0.056, 95% CI [−0.160, 0.053]), whereas both indirect effects were statistically significant, albeit with small-to-moderate effect sizes. These findings contribute to understanding the associated mechanisms of academic difficulties in college students with elevated ADHD symptoms, particularly highlighting the dual pathways of motivation and mind-wandering. The study offers tentative theoretical implications and suggests directions for future intervention research (e.g., motivational reinforcement, attentional training); given the small-to-moderate absolute magnitudes of the indirect effects, these practical implications should be interpreted with caution pending replication.

## 1 Introduction

Attention Deficit/Hyperactivity Disorder (ADHD) is a common neurodevelopmental disorder in childhood, characterized by difficulties in attention, hyperactivity, and impairments in social functioning. Low academic achievement is a prevalent functional impairment in individuals with ADHD (Sonuga-Barke et al., 2013). Adult ADHD affects a substantial proportion of adults and is frequently underdiagnosed (Kessler et al., 2006), and in China ADHD prevalence among children and adolescents is also considerable (Wang et al., 2017). Adult ADHD is often more challenging to diagnose, with lower consultation rates compared to children (Cortese and Coghill, 2018), and the struggles of adult students with ADHD are frequently overlooked (Liu et al., 2023). It is crucial to recognize that students with elevated ADHD symptoms of all ages are at risk for academic difficulties and failure (Augustin, 2014; Faraone et al., 1993).

Most research on ADHD-related academic difficulties focuses on younger children, with limited studies on college students, thereby neglecting the developmental nature of ADHD and the hidden severity of adult symptoms. College students with elevated ADHD symptoms face significant academic and social challenges, often compounded by a lack of understanding of their symptoms and delayed or inadequate medical intervention.

Efficient learning strategies are critical for academic success and learning efficiency (Yue et al., 2024). However, individuals with ADHD often struggle to develop or effectively use such strategies (Simon-Dack et al., 2016). This raises the question: do the academic difficulties experienced by individuals with elevated ADHD symptoms stem, in part, from a lack of awareness and use of efficient learning strategies? To answer this question, the present study investigates the relationship between ADHD symptoms and the utilization of efficient learning strategies, examining the parallel mediating roles of motivation and mind-wandering in this relationship.

## 2 Theoretical model derivation

### 2.1 Efficient learning strategies

Efficient learning strategies are evidence-based methods that have been empirically shown to enhance long-term retention and academic performance; the present study focuses on two such strategy types—spaced learning and self-testing—which are among the most robustly supported in the cognitive psychology literature and were assessed with four dedicated items in the strategy-use measure. Spaced learning and self-testing are proven to be well-established strategies that not only help consolidate learned material but also improve learning ability, significantly enhancing long-term retention and memory (Augustin, 2014). Spaced learning has been shown to enhance memory (Feng et al., 2019), with its effects lasting longer and being more profound than simple repetition (Ebbinghaus, 2013). Self-testing, another crucial strategy, is equally important for long-term memory and comprehension, helping to assess knowledge acquisition while reinforcing retention (Roediger III and Karpicke, 2006). It has been shown that self-testing is more effective than other methods, such as note-taking or summarizing, especially compared to rereading (Rummer et al., 2017). Furthermore, self-testing enhances understanding of new knowledge and strengthens memory retention (Rowland, 2014).

It is important to note that spaced learning and test-based strategies are not completely distinct but often influence each other. Regular testing encourages learners to adopt spaced learning by organizing study time and planning their study schedule, avoiding the “cramming” approach before exams. Spaced learning drives learners to retrieve and recall knowledge by increasing the number of retrieval attempts, which helps enhance mastery of the content and promotes a deeper understanding and long-term retention of learned material (Bjork, 1988).

### 2.2 ADHD symptoms and use of efficient learning strategies

ADHD symptoms include lack of learning motivation, self-perception biases, frequent mind-wandering, executive dysfunction, and difficulties with planning and memory organization. Spaced learning strategies demand that learners formulate and rigorously execute a well-structured learning plan, while self-testing strategies require learners to respond well to external stimuli. Moreover, the successful implementation of efficient learning strategies is dependent on learners’ self-concept and positive motivation (Zhang et al., 2017). Based on this, the present study examines whether students with elevated ADHD symptoms suffer from a lack of efficient learning strategies and explores the underlying reasons.

ADHD presents differently in adulthood than in childhood. Adult symptoms are often more latent and subthreshold: diagnosis rates among adults seeking treatment are approximately 2.5% compared with approximately 5.3% in children, and adults with elevated symptoms are less likely to be diagnosed or to attend treatment (Cortese and Coghill, 2018; Rajaprakash and Leppert, 2022). In college settings, academic demands shift toward self-regulated learning with reduced external structure, which is precisely the environment in which motivational and attentional difficulties are most consequential. These developmental and contextual considerations motivate our focus on college students with elevated ADHD symptoms.

Several factors affect the use of efficient learning strategies among individuals with elevated ADHD symptoms, with unclear internal mechanisms. This study focuses on two main conditions: lack of motivation and mind-wandering. These factors are explored as predictors of learning strategy use by college students with elevated ADHD symptoms. Mind-wandering, a key mechanism explaining many ADHD symptoms, is associated with impairments in cognitive functions (Mowlem et al., 2019). Neuroscientific studies have identified dysregulated neural activity as the pathological basis of ADHD. Research indicates that mind-wandering is a primary deficit contributing to this dysregulation, directly impacting attention and executive function, which are crucial for using learning strategies effectively (Bozhilova et al., 2018). Thus, mind-wandering could be a central factor limiting the efficient use of learning strategies in individuals with elevated ADHD symptoms. Additionally, individuals with elevated ADHD symptoms often struggle with executing instructions, leading to low motivation in learning activities (Woods et al., 2020). The inability to use efficient learning strategies can stem from the lack of positive learning motivation (Simon-Dack et al., 2016), suggesting that motivation plays a key role in the effective use of learning strategies for individuals with elevated ADHD symptoms.

We deliberately focus on motivation and mind-wandering rather than broader constructs such as general executive function or self-regulation for two reasons. First, both constructs map onto the two most prominently affected domains in ADHD—motivational deficits and attentional control—and each has a theoretically separable pathway to strategy use: motivation determines the initiation and persistence of strategy engagement (Deci and Ryan, 2000), whereas mind-wandering captures a specific attentional failure mechanism that disrupts ongoing strategy execution (Bozhilova et al., 2018). Second, both are amenable to targeted intervention in educational settings. This theoretical differentiation motivates the parallel-mediation model tested below.

### 2.3 The mediating role of learning motivation in ADHD symptoms and use of efficient learning strategies

#### 2.3.1 ADHD symptoms and motivation to learn

Learning motivation refers to the internal drive that initiates, directs, and sustains learning behaviors (Deci and Ryan, 2000). Learning motivation is a complex, multidimensional concept that plays a critical role in enhancing learning (Wulf and Lewthwaite, 2016; Ilieva and Farah, 2019). However, individuals with elevated ADHD symptoms exhibit deficiencies in stimulating internal motivation and processing external stimuli compared to the general population, which presents barriers to generating and maintaining motivation (Zawiślak et al., 2023). Emotional regulation disorders further exacerbate this challenge, influencing the development of motivation in individuals with elevated ADHD symptoms (Cook and Artino, 2016; Dela Rosa and Bernardo, 2013). The frontal lobe’s functioning, which is critical for motivation generation and maintenance, is impaired in individuals with elevated ADHD symptoms, leading to difficulties in stimulating and sustaining learning behaviors (Bokor and Anderson, 2014; Magnus et al., 2024). Therefore, the deficiency in motivation is a significant factor contributing to the learning difficulties observed in individuals with elevated ADHD symptoms (Antunes et al., 2022; Rosch and Mostofsky, 2019). Based on these findings, we propose Hypothesis 1: There is a negative correlation between ADHD symptoms and learning motivation.

#### 2.3.2 Motivation and the use of efficient learning strategies

Lack of motivation is a major cause of poor strategy awareness and underuse (Sella et al., 2019; Simon-Dack et al., 2016). This lack of motivation directly limits the development and implementation of efficient learning strategies, further exacerbating executive dysfunction and control difficulties (Orhan et al., 2023). Motivation is essential for students to engage with and explore efficient learning strategies (Dunlosky and Thiede, 1998; Tao et al., 2019). It also guides the selection and application of these strategies, making students more proactive in using them and maintaining an optimistic attitude toward self-testing (Wulf and Lewthwaite, 2016; Ma, 2024). Motivation also plays a crucial role in reinforcing learning by adopting effective strategies to achieve academic goals (Bandhu et al., 2024). Thus, we propose Hypothesis 2: There is a positive correlation between motivation and the use of efficient learning strategies.

#### 2.3.3 The mediating role of learning motivation

Individuals with elevated ADHD symptoms typically exhibit low levels of motivation, as evidenced by task avoidance and reliance on external support (Simon-Dack et al., 2016). This lack of motivation leads to limited flexibility in developing and applying learning strategies, contributing to the underuse of these strategies (Colomer et al., 2017; Dekkers et al., 2017). Longitudinal studies have shown that individuals with ADHD or elevated ADHD symptoms experience a significant decrease in motivation as task duration increases, especially when feedback or rewards are delayed. Individuals with elevated ADHD symptoms also show abnormal functioning in dopamine neural circuits and the amygdala, further impairing motivation and executive functioning (Scheres and Solanto, 2021). Based on these findings, we hypothesize that insufficient motivation is a key factor influencing the use of efficient learning strategies in individuals with elevated ADHD symptoms. Thus, Hypothesis 3 is proposed: ADHD symptoms are negatively associated with motivation, which in turn is positively associated with the use of efficient learning strategies, constituting an indirect pathway.

### 2.4 The mediating role of mind-wandering in ADHD symptoms and use of efficient learning strategies

#### 2.4.1 ADHD symptoms and mind-wandering

Mind-wandering refers to the shift of attention from the primary task toward internal, task-unrelated thoughts (Seli et al., 2015). Mind-wandering can be classified into intentional and spontaneous types, both of which are thought to result from unbalanced internal regulatory processes (Seli et al., 2015). Spontaneous mind-wandering, in particular, explains many symptoms and deficits in ADHD (Seli et al., 2015; Mowlem et al., 2019). Neuroscientific studies suggest that impaired monoamine neurotransmitter pathways contribute to ADHD’s pathology. Mind-wandering in ADHD is linked to abnormalities in neurotransmitter functioning, such as dopamine and norepinephrine, leading to disruptions in attention and planning (Cheng et al., 2018). MRI studies show that individuals with ADHD have reduced grey and white matter volumes, with grey matter abnormalities affecting cognitive functions like attention and planning (Wu et al., 2017). These impairments result in individuals with elevated ADHD symptoms being more prone to mind-wandering than their peers. Therefore, we propose Hypothesis 4: There is a positive correlation between ADHD symptoms and mind-wandering in college students.

#### 2.4.2 Mind-wandering and efficient learning strategies

When the mind wanders, the brain’s efficiency in processing information decreases, disrupting learning and the ability to apply strategies. Mind-wandering interferes with the brain’s self-monitoring of learning, further hindering strategy use. Clinical studies show that individuals in a state of mind-wandering show abnormalities in brain regions like the frontal lobe and basal ganglia, leading to executive dysfunction and poor problem-solving abilities (Anderson, 2002; Krain and Castellanos, 2006). As a result, individuals who experience frequent mind-wandering find it difficult to plan and execute learning strategies, especially spaced learning. Therefore, we hypothesize 5: There is a negative correlation between mind-wandering and the use of efficient learning strategies.

#### 2.4.3 The mediating role of mind-wandering

Individuals with elevated ADHD symptoms often exhibit deficits in learning strategies (e.g., organizational skills, study skills, and time management) (Dehili et al., 2017). Mind-wandering significantly affects the use of efficient learning strategies in individuals with ADHD, contributing to poor academic performance (Sella et al., 2019). Follow-up studies have shown that executive dysfunction and mind-wandering predict learning approaches in individuals with elevated ADHD symptoms, which indirectly influences academic performance (Morsanyi et al., 2018). Inattention, as a key factor in learning strategy development, exacerbates the underuse of strategies as mind-wandering duration increases (Shelton et al., 2019). Based on these findings, we propose Hypothesis 6: ADHD symptoms are positively associated with mind-wandering, which in turn is negatively associated with the use of efficient learning strategies, constituting an indirect pathway.

#### 2.4.4 Summary of the proposed model

Current research on ADHD and academic success in adults is limited, making early intervention challenging. Although some interventions have shown promise (Fredriksen and Peleikis, 2016), these studies often fail to control for confounding variables. Most ADHD-related academic research has focused on younger children, neglecting the developmental nature of ADHD and the hidden symptoms in adults. This study investigates ADHD symptoms and the use of efficient learning strategies among college students, examining the parallel mediating roles of motivation and mind-wandering. By analyzing how motivation and mind-wandering are associated with learning strategies, this study aims to provide a more accurate understanding of academic performance in college students with elevated ADHD symptoms and to inform strategies for improving learning outcomes in this population.

## 3 Methodology

### 3.1 Subjects of study

A convenience sampling method was employed to select participants from a university in Sichuan Province. A total of 919 questionnaires were distributed, with 730 valid responses returned, yielding an effective response rate of 79.43%. After excluding questionnaires that did not meet the required criteria (e.g., incomplete information, excessively short response times, or patterned answers), the final dataset consisted of 730 valid questionnaires. Among the participants, 239 were male (32.7%) and 491 were female (67.3%), with a mean age of 19.95 years (SD = 1.03, range 18–25; *N* = 714 with valid age data). Participants represented a diverse range of academic disciplines, including medicine, law, management, science, and engineering.

### 3.2 Measurement variables and tools

#### 3.2.1 ADHD symptoms

The Adult ADHD Self-Report Scale (ASRS) is a World Health Organization instrument for assessing DSM-defined ADHD symptoms in adults (Kessler et al., 2005). The present study used the complete ASRS Symptom Checklist, comprising 18 items spanning two symptom dimensions—inattention and hyperactivity/impulsivity—scored on a 5-point Likert scale ranging from “never” to “always.” This 18-item Symptom Checklist is distinct from the widely used 6-item ASRS Screener, which is a validated subset of the same item pool designed for rapid case-finding rather than for dimensional symptom measurement (Kessler et al., 2005). Because the present study aimed to capture ADHD symptom severity as a continuous dimensional construct rather than to conduct case-finding screening, the full 18-item Symptom Checklist, rather than the 6-item Screener, was administered, and the continuous ASRS total score (the sum of all 18 items, possible range 0–72) was used as the indicator of ADHD symptom severity in all substantive analyses. The ASRS was also employed as the calibration tool to assess the validity of the Chinese version of the 4FMW (mind-wandering) scale. The Cronbach’s alpha coefficient for the full scale was 0.908, indicating strong internal consistency, and the scale demonstrated good construct validity.

Representative example items are: inattention–“When you do a boring or repetitive task, do you have difficulty maintaining your focus?”; hyperactivity/impulsivity–“When you must sit still for a long time, do you often fidget or move your hands and feet?” The ASRS was originally developed and validated by Kessler et al. (2005) against clinician-administered diagnostic interviews, and the Chinese version used here has demonstrated comparable reliability and construct validity in Chinese university samples.

#### 3.2.2 Motivation to learn

Following Self-Determination Theory (SDT; Deci and Ryan, 2000), academic motivation was conceptualized along the self-determination continuum ranging from amotivation to intrinsic motivation. The Chinese Academic Motivation Scale, revised by Bo Zhang et al. (2016), was used to assess academic motivation. The Chinese Academic Motivation Scale, adapted from Vallerand et al. (1992) original Academic Motivation Scale, was used to assess academic motivation. This scale includes seven dimensions: amotivation, external regulation, introjected regulation, identified regulation, intrinsic motivation–knowledge, intrinsic motivation–accomplishment, and intrinsic motivation–stimulation, which represent successive points on the SDT continuum. The original scale uses a 5-point response format; for the present study, the items were adapted to a 5-point scale, with scores ranging from 1 (“not at all”) to 5 (“very much”). The scores for each dimension were averaged, with higher scores reflecting stronger motivation in that specific category. In this study, the Cronbach’s alpha coefficient for the scale was 0.837, and it demonstrated good structural validity.

Representative example items (verbatim from the source questionnaire) are: amotivation -- “Honestly, I don’t know; I really feel that I am wasting my time in school”; external regulation -- “Because I want to get a more prestigious job (e.g., manager, engineer) later on”; introjected regulation – “To prove to myself that I am capable of completing my college education”; identified regulation -- “Because I think that college education will help me better prepare for the job that I like”; intrinsic motivation to know -- “Because I feel happy and satisfied while learning new things”; intrinsic motivation toward accomplishment -- “For the enjoyment that I feel when I do better than the last time in my studies”; intrinsic motivation to experience stimulation -- “Because for me, school is fun.” The original English scale was developed by Vallerand et al. (1992) and the Chinese version was validated by Zhang et al. (2016), who reported a well-fitting seven-factor structure and satisfactory subscale reliability in Chinese samples.

#### 3.2.3 Mind-wandering

Following Seli et al. (2015), mind-wandering was defined as attention shifting away from the primary task toward internal thoughts, with deliberate and spontaneous forms distinguished; the present study focuses on spontaneous mind-wandering experiences as captured by the 4FMW. The Four Factors of Mind Wandering Questionnaire (4FMW; Lopez et al., 2023) was used to measure mind-wandering. The questionnaire consists of 16 items across four dimensions: failure in social interaction, failure in interaction with objects, unawareness, and inattention. The original scale uses a 5-point Likert scale. The Cronbach’s alpha coefficient for the total scale was 0.908, indicating strong internal consistency, and the scale showed good construct validity.

Representative example items are: failure in social interaction -- “Realizing you forgot part of a conversation because you were not focused”; failure in interaction with objects -- “Picking up the wrong thing (e.g., taking wine from the fridge instead of milk)”; unawareness -- “Being unaware of what is happening around you”; inattention -- “Unable to concentrate on the text being read; must re-read the same content.” The 4FMW was developed and validated by Lopez et al. (2023), who established its four-factor structure and reported acceptable reliability and construct validity.

#### 3.2.4 Efficient learning strategies

Strategy use was assessed with a dedicated strategy-use measure that operationalized “efficient learning strategies” as four specific items capturing self-testing and spaced-learning practices. “When studying, I test myself (e.g., by actively recalling what I have learned, reciting, writing from memory, or doing practice exercises)”; “During the one or two weeks before an exam, I review a portion of the material each day, rather than cramming right before the exam”; “I make a study plan in advance and study according to it”; “I like in-class quizzes.” Items were rated on a 5-point Likert scale (1–5), and the four items were summed to a total score (range 4–20). In the present sample (*N* = 730), the total score had M = 12.518, SD = 2.756, and a Cronbach’s α of 0.751, indicating acceptable internal consistency for a four-item scale.

### 3.3 Statistical analysis

Data were analyzed using SPSS 27.0 and AMOS 23.0. Descriptive statistics and zero-order correlations were computed in SPSS. The hypothesized parallel-mediation model was estimated as a latent-variable structural equation model (SEM) in AMOS: mind-wandering (F1, four 4FMW factor scores as indicators), learning motivation (F2, six SDT motivational subscale scores as indicators; amotivation was excluded as the low pole of the SDT continuum), efficient learning strategies (four strategy items as indicators), and ADHD symptoms (ASRS total score as a single indicator). Standardized indicator loadings ranged from 0.48 to 0.89. Because the freely estimated residual variance of the single ADHD indicator was negative (a Heywood case, e22 = −12.458; AMOS reported that the solution was not admissible), the residual variance of this indicator was fixed to zero, treating the ASRS total as a perfectly measured composite; for a single-indicator latent variable, this is mathematically equivalent to entering the observed total score into the model (Hayduk and Littvay, 2012). The re-estimated model converged to an admissible solution without any negative variances, and the substantive estimates were virtually identical to the original solution. We therefore report the single-indicator specification as the primary model: it yields an admissible, Heywood-free solution, and the substantive conclusions about the mediation structure are unaffected. The indirect effects were tested with the non-parametric percentile bootstrap method with bias correction, drawing 5,000 samples to compute the 95% confidence intervals for the mediation effects (Fang et al., 2014). All reported path coefficients and indirect/direct effects are standardized (*β*).

Because all measures were self-report, Harman’s single-factor test was conducted on all 66 scale items (18 ASRS items + 16 4FMW items + 28 Academic Motivation Scale items + 4 efficient-learning-strategies items) to examine common method bias. The first unrotated factor accounted for 24.54% of the total variance (eigenvalue 16.20), well below the conventional 40% threshold, and 10 factors had eigenvalues greater than 1, indicating that common method bias is unlikely to seriously threaten the validity of the results.

## 4 Analyses and results

### 4.1 Descriptive statistical analyses

Table 1 illustrates that ADHD symptoms were significantly negatively correlated with the use of efficient learning strategies (*r* = −0.259, *p* < 0.001). Furthermore, ADHD symptoms showed a significant negative correlation with learning motivation (*r* = −0.152, *p* < 0.001) and a significant positive correlation with mind-wandering (*r* = 0.682, *p* < 0.001). Additionally, mind-wandering was significantly negatively correlated with the use of efficient learning strategies (*r* = −0.224, *p* < 0.001), while learning motivation was positively correlated with the use of efficient learning strategies (*r* = 0.573, *p* < 0.001). These results support the mediation model presented in Figure 1.

**Table 1:** Descriptive statistics and correlations among the main variables.

Variables	M	SD	1	2	3	4
1. ADHD Symptoms	43.841	8.609	—			
2. Mind-wandering	41.047	9.513	0.682***	—		
3. Learning motivation	98.078	13.923	−0.152***	−0.079*	—	
4. Efficient learning strategies	12.518	2.756	−0.259***	−0.224***	0.573***	—

*N* = 730.*p < 0.05, **p < 0.01, ***p < 0.001 (two-tailed).

**Figure 1:**
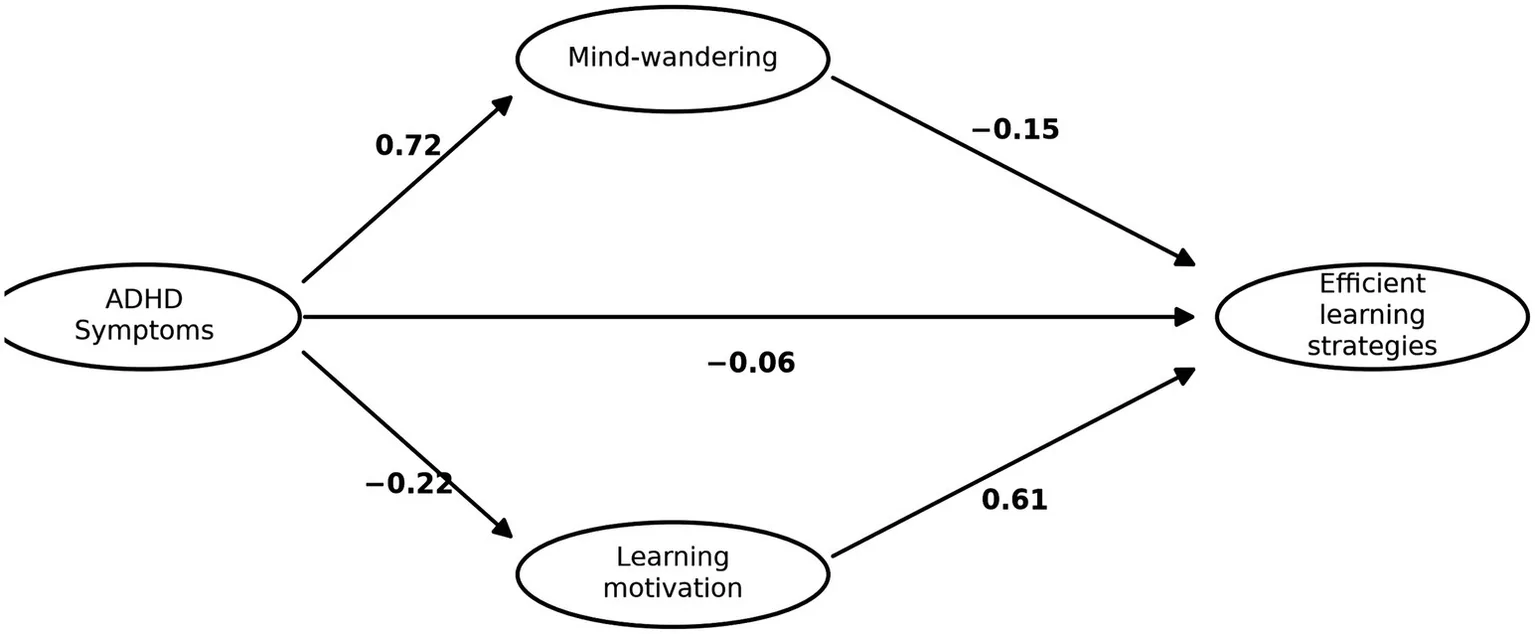
Parallel mediation of mind-wandering and learning motivation between ADHD symptoms and efficient learning strategies. All path coefficients shown are standardized estimates. Standardized partial-path coefficients (e.g., ADHD Symptoms → learning motivation = −0.22) differ from the bivariate zero-order correlations reported in Table 1 (e.g., *r* = −0.152).

### 4.2 Mediated effects test

All analyses were conducted in AMOS using the latent-variable model described in Section 3.3. To address the female-skewed sample composition (67.3% female), we conducted supplementary gender sensitivity analyses (reported below). Mind-wandering and learning motivation were incorporated as mediators, and the non-parametric percentile bootstrap method with bias correction was applied, drawing 5,000 samples to compute the 95% confidence intervals for the mediation effects (Hayes, 2013). The results showed that the overall model fit was acceptable: χ^2^(79) = 379.03, *p* < 0.001, χ^2^/df = 4.80; CFI = 0.958, TLI = 0.943, GFI = 0.947, AGFI = 0.929; RMSEA = 0.072 (90% CI [0.065, 0.080]); SRMR = 0.054. All indices met conventional thresholds, except that the upper bound of the RMSEA confidence interval approached the 0.08 threshold; we therefore interpret the model results with appropriate caution.

Gender sensitivity analyses. Gender (male = 1) was weakly associated with ADHD symptoms (*r* = −0.08, *p* = 0.036) and mind-wandering (*r* = −0.15, *p* < 0.001), but unrelated to learning motivation (*r* = −0.06, *p* = 0.10) and efficient learning strategies (*r* = −0.01, *p* = 0.74). Re-estimating the parallel-mediation model with gender entered as a covariate produced a virtually identical pattern (all path coefficients shifted by <0.01 in absolute value). Formal moderation tests -- gender × ADHD interactions predicting the mediators, and gender x mediator interactions predicting the outcome -- were all non-significant (ps = 0.19–0.98), indicating that gender did not moderate the mediation pathways. We therefore report the gender-unadjusted primary model and note that the conclusions are robust to participants’ gender.

The results of the mediation analysis are shown in Table 2. Two mediation paths were identified: Path 1, ADHD symptoms → mind-wandering → efficient learning strategies (standardized effect (*β*) = −0.107, 95% CI [−0.198, −0.027]); Path 2, ADHD symptoms → learning motivation → efficient learning strategies (standardized effect (*β*) = −0.130, 95% CI [−0.196, −0.061]). Both mind-wandering and learning motivation acted as negative mediators. The 95% confidence intervals for both paths did not include 0, indicating that the mediation effects of both paths were significant. The direct effect of ADHD symptoms on efficient learning strategies was not significant (standardized effect (*β*) = −0.056, 95% CI [−0.160, 0.053]), as the 95% confidence interval for this path included 0. Thus, the direct effect was non-significant while both indirect effects were significant; we describe this pattern as consistent with full mediation in the statistical sense, while noting that this conclusion is model-dependent and does not rule out unmeasured mechanisms. Mind-wandering accounted for 45.2% of the total indirect effect, while learning motivation accounted for 54.8%. The comparison of the two indirect effects was not significant (standardized effect (*β*) = 0.023, 95% CI [−0.094, 0.131]), indicating that the two pathways did not differ significantly.

**Table 2:** Results of the analysis of indirect effects.

Path	*β*	SE	95% CI [lower, upper]	*p*
ADHD Symptoms → Mind-wandering → Learning strategies	−0.107	0.043	[−0.198, −0.027]	0.014
ADHD Symptoms → Learning motivation → Learning strategies	−0.130	0.035	[−0.196, −0.061]	<0.001
Total indirect effect	−0.237	0.054	[−0.340, −0.128]	<0.001
Direct effect	−0.056	0.054	[−0.160, 0.053]	0.300
Contrast of indirect effects	0.023	0.057	[−0.094, 0.131]	0.685

All effects are standardized indirect/direct effects (*β*); bootstrap 95% confidence intervals based on 5,000 samples (AMOS). Standard errors (SE) and p values (Wald test, *β*/SE) are derived from the same bootstrap distribution.

It should be noted that the absolute magnitudes of the standardized indirect effects were small-to-moderate (*β* = −0.107 for the mind-wandering pathway and *β* = −0.130 for the learning-motivation pathway). Statistical significance (bootstrap CIs excluding zero) should therefore be distinguished from practical importance.

## 5 Discussion

### 5.1 Relationship between ADHD symptoms and efficient learning strategies in college students

The study found a significant relationship between ADHD symptoms and the use of efficient learning strategies; however, ADHD symptoms alone could not predict the use of such strategies. These findings are consistent with previous research, which suggests a correlation between ADHD symptoms and efficient learning strategies usage (DuPaul et al., 2021). Building on previous research, this study reveals that ADHD symptoms are statistically associated with the use of efficient learning strategies through two mediators: learning motivation and mind-wandering, with both mind-wandering and learning motivation acting as negative mediators.

Previous research on ADHD has predominantly focused on children and adolescents, often treating ADHD as a disorder unique to childhood. This perspective has, to some extent, overlooked the disorder’s characteristics and complexities in adulthood. This limitation is mainly due to the more latent nature of ADHD symptoms in adults, which manifest in lower diagnosis rates (approximately 2.5%) compared to children (approximately 5.3%), higher diagnostic difficulty in adults, and lower attendance rates for treatment (Cortese and Coghill, 2018; Rajaprakash and Leppert, 2022). Consequently, there is a notable lack of academic-related research on adults with elevated ADHD symptoms. Based on previous studies on learning strategies and academic performance in individuals with elevated ADHD symptoms, this research focuses on analyzing the negative associations of insufficient learning motivation and frequent mind-wandering with the use of efficient learning strategies. These findings may provide an educational and psychological explanation for the poor academic performance often observed in individuals with elevated ADHD symptoms.

### 5.2 Mediating role of learning motivation

The results confirmed Hypotheses 1, 2, and 3, indicating that learning motivation mediates the relationship between ADHD symptoms and efficient learning strategies. Individuals with poor executive functions tend to have weaker ability to follow instructions, leading to a lack of learning motivation during academic tasks (Woods et al., 2020). On the other hand, individuals with high learning motivation often maintain intrinsic interest in their studies, driving them to adopt a flexible and diverse set of strategies that adapt to environmental demands while maintaining motivation (Bokor and Anderson, 2014). Furthermore, learning motivation promotes the exploration, practice, and long-term implementation of learning strategies (Simon-Dack et al., 2016), acting as the primary driver in initiating and sustaining lengthy and often challenging learning processes.

Additionally, based on Deci and Ryan’s Self-Determination Theory (Deci and Ryan, 2000), prior studies have suggested that learning motivation plays a key role in learners’ engagement and the formulation of learning strategies by fostering a sense of self-efficacy (Babenko and Oswald, 2019; Wu et al., 2020). Once learners have clear learning goals, they are more likely to select appropriate strategies to achieve those goals (Kim et al., 2014). However, due to significant barriers in meeting basic learning task demands, individuals with elevated ADHD symptoms often experience impaired internalization of their drive, leading to insufficient learning motivation (Morsink et al., 2022). This pattern may potentially involve mechanisms not directly measured in the present study, such as reduced satisfaction derived from completing tasks; within the measured constructs, this combination of negative factors results in a lack of enthusiasm for academic tasks, preventing the formulation of long-term learning goals, and ultimately diminishing the effectiveness and flexibility of their learning strategies.

### 5.3 Mediating role of mind-wandering

The results confirmed Hypotheses 4, 5, and 6, indicating that mind-wandering mediates the relationship between ADHD symptoms and efficient learning strategies. Learners with elevated ADHD symptoms often experience high frequencies of mind-wandering, making it difficult to focus on current learning tasks. This reduces the efficiency of information processing in the brain and weakens the coordination between related learning functions, ultimately impairing their ability to construct academic and goal-related cognition, which in turn impacts the formulation and use of learning strategies (Bozhilova et al., 2018; Merrill et al., 2022). Therefore, the frequency of mind-wandering is closely associated with cognitive function deficits. Additionally, mind-wandering can weaken an individual’s metacognitive monitoring abilities, making it difficult for them to effectively assess and adjust learning strategies. This metacognitive deficiency may lead to improper selection and execution of learning strategies, further affecting academic performance (McVay and Kane, 2010). Thus, mind-wandering not only directly interferes with task performance in individuals with elevated ADHD symptoms but also indirectly reduces the ability to use efficient learning strategies (such as self-testing and spaced repetition).

### 5.4 Parallel mediation role of learning motivation and mind-wandering between ADHD symptoms and efficient learning strategies use

Both learning motivation and mind-wandering were found to account for the relationship between ADHD symptoms and the use of efficient learning strategies: the direct effect was non-significant while both indirect effects were significant. Full mediation in this statistical sense is model-dependent and does not rule out unmeasured mechanisms. This phenomenon can be attributed to the fact that insufficient learning motivation and increased mind-wandering directly lower the ability of individuals with elevated ADHD symptoms to develop and use efficient learning strategies (Colomer et al., 2017; Sella et al., 2019). Moreover, these factors may affect the formulation of learning goals and the planning of academic tasks through other pathways, including reduced learning interest, impaired response inhibition, interference with emotional regulation, decreased tolerance for delayed rewards, and weakened working memory, which further hinder the effectiveness and flexibility of learning strategy use (McVay and Kane, 2010; Sibley et al., 2019).

Learning motivation can be divided into intrinsic and extrinsic motivation. Intrinsic motivation is considered a long-term, enduring driver that maintains learners’ interest in academic tasks or satisfaction of psychological needs. Extrinsic motivation reflects the influence of social demands and educational environments on learners, driving them to engage in learning behaviors to obtain rewards or avoid punishment in response to external pressures (Bandhu et al., 2024; Morsink et al., 2022). The interplay between intrinsic and extrinsic motivation fosters the development and use of learning strategies, thereby enhancing academic achievement. Mind-wandering, as another major disruptive factor in the learning process, primarily interferes with working memory and learning memory, leading to the disruption of multiple learning and executive functions, further weakening the effective planning and execution of learning strategies (Bozhilova et al., 2018). Based on this discussion, we may explain the observed pattern of results—statistically significant indirect effects through both mediators with no significant difference between them—and the lack of a significant direct effect of ADHD symptoms on efficient learning strategies.

### 5.5 Significance and limitations

This study focuses on ADHD symptoms within the college student population, aiming to explore the developmental characteristics of ADHD symptoms and their latent presentation in adulthood from an individual internal-factors perspective. It systematically investigates the relationship between ADHD symptoms and the use of efficient learning strategies. The research seeks to uncover the potential deficiencies and mechanisms of learning strategy use in individuals with elevated ADHD symptoms, providing a theoretical basis for promoting the application of efficient learning strategies, improving learning efficacy, optimizing academic performance, and supporting individual self-growth and holistic development. The theoretical contribution of this study is a parallel-mediation framework for dimensional ADHD symptomatology in college samples that integrates Self-Determination Theory and the mind-wandering perspective; its practical implications point to motivational reinforcement strategies and attention training as complementary routes for optimizing learning strategy use among students with elevated ADHD symptoms. Additionally, in response to the limitations of previous studies in controlling confounding variables (Sroubek et al., 2013), this study systematically measures college students’ ADHD symptom levels, learning motivation, and mind-wandering, offering a deeper analysis of the pathways through which ADHD symptoms are associated with the use of efficient learning strategies. This research provides empirical support for intervention strategies related to learning problems in students with elevated ADHD symptoms and lays the foundation for developing scientific and systematic practical solutions.

However, this study has several limitations. First, in terms of geographic and demographic scope, the research was conducted at a single university in Sichuan, which may limit the generalizability of the findings and introduce potential biases. Future studies may consider expanding the survey sample, implementing a mechanism for identifying and handling invalid questionnaires, and improving the accuracy of data collection. Second, there are limitations in controlling for the gender variable. Previous studies have indicated gender differences in the prevalence and symptom expression of ADHD (e.g., 7.7% for boys and 3.4% for girls). Boys with ADHD often exhibit externalizing issues such as hyperactivity, impulsivity, and aggression, while girls with ADHD tend to show internalizing issues, including psychosomatic disorders (Huang and Lin, 2020). These gender differences may affect the use of learning strategies to some extent. Therefore, future studies may consider incorporating gender-based subgroup models to broaden the scope of the research. Third, all measures were self-report; although Harman’s single-factor test suggested that common method bias is not a serious concern, shared-method variance cannot be fully excluded. Fourth, the indirect effects identified were small-to-moderate in absolute standardized magnitude (*β* = −0.107 for the mind-wandering pathway and *β* = −0.130 for the learning-motivation pathway); their practical significance should therefore be interpreted cautiously, and replications with larger or longitudinal samples are needed before strong practical claims can be made. Fifth, ADHD symptoms were modeled as a single-indicator latent variable using the ASRS total score, which assumes the composite is measured without error; this is a pragmatic identification choice rather than an optimal psychometric solution, and the significance of the mind-wandering pathway was found to be sensitive to this specification, whereas the motivation pathway and the overall mediation pattern were robust across specifications. Sixth, the construct of “efficient learning strategies” was operationalized as a four-item composite (items 6.6, 6.7, 6.8, and 6.16) targeting self-testing and spaced-learning practices, with a Cronbach’s α of 0.751. Although the internal consistency is acceptable for a short scale, the narrow item set restricts content breadth and may underrepresent the broader repertoire of efficient learning strategies; future studies should consider expanding the item pool while retaining the theoretical focus on retrieval practice and spacing.

Additionally, this study utilized a cross-sectional design. Despite gathering rich data through large-scale surveys, the time-point nature of the data limits the ability to draw causal inferences between variables. Although this study built a theoretical model based on existing empirical research and employed statistical methods to test hypotheses, the generalizability of the findings still requires further validation through longitudinal tracking or experimental designs.

Nonetheless, by constructing parallel mediation models, this study provides initial insights into the complex mechanisms linking ADHD symptoms, learning motivation, mind-wandering, and the use of efficient learning strategies. Notwithstanding these limitations, the present study offers preliminary empirical leads on the mechanisms linking ADHD symptoms, motivation, mind-wandering, and efficient learning-strategy use. Because the standardized indirect effects are small-to-moderate, we caution against strong practical claims and recommend replication in larger or longitudinal samples before firm conclusions are drawn.

## Generative AI disclosure

During the preparation and revision of this manuscript, the authors used WorkBuddy (an AI-powered writing assistant) to support English language editing. The authors reviewed and edited all AI-generated suggestions and take full responsibility for the content of this publication.

## Data Availability

The deposited data are aggregated subset variables supporting the findings and exclude all raw item responses; the complete dataset is available from the corresponding author upon reasonable request.
